# Impact of single-incision laparoscopic cholecystectomy (SILC) versus conventional laparoscopic cholecystectomy (CLC) procedures on surgeon stress and workload: a randomized controlled trial

**DOI:** 10.1007/s00464-015-4332-5

**Published:** 2015-07-21

**Authors:** Amro M. Abdelrahman, Juliane Bingener, Denny Yu, Bethany R. Lowndes, Amani Mohamed, Andrea L. McConico, M. Susan Hallbeck

**Affiliations:** Robert D. and Patricia E. Kern Center for the Science of Health Care Delivery, Mayo Clinic, Rochester, MN USA; Department of Surgery, Mayo Clinic, Rochester, MN USA; Division of Health Care Policy and Research, Department of Health Sciences Research, Mayo Clinic, 200 First Street SW, Rochester, MN 55905 USA

**Keywords:** Surgeon, Laparoscopy, SILC, Workload, Surg-TLX, Stress

## Abstract

**Introduction:**

Single-incision laparoscopic cholecystectomy (SILC) may lead to higher patient satisfaction; however, SILC may expose the surgeon to increased workload. The goal of this study was to compare surgeon stress and workload between SILC and conventional laparoscopic cholecystectomy (CLC).

**Methods:**

During a double-blind randomized controlled trial comparing patient outcomes for SILC versus CLC (NCT0148943), surgeon workload was assessed by four measures: surgery task load index questionnaire (Surg-TLX), maximum heart rate, salivary cortisol level, and instruments usability survey. The maximum heart rate and salivary cortisol levels were sampled from the surgeon before the random assignment of the surgical procedure, intraoperatively after the cystic duct was clipped, and at skin closure. After each procedure, the surgeon completed the Surg-TLX and an instrument usability survey. Student’s *t* tests, Wilcoxon rank sum test, and Kruskal–Wallis nonparametric ANOVAs on the dependent variables by the technique (SILC vs. CLC) were performed with *α* = 0.05.

**Results:**

Twenty-three SILC and 25 CLC procedures were included in the intent-to-treat analysis. No significant differences were observed between SILC and CLC for patient demographics and procedure duration. SILC had significantly higher post-surgery surgeon maximum heart rates than CLC (*p* < 0.05). SILC also had significantly higher mean change in the maximum heart rate between during and post-procedure (*p* < 0.05) than CLC. Salivary cortisol level was significantly higher during SILC than CLC (*p* < 0.01). Awkward manipulation of the instruments and limited fine motions were reported significantly more frequently with SILC than CLC (*p* < 0.01). In the surgeon-reported Surg-TLX, subscale of physical demand was significantly more demanding for SILC than CLC (*p* < 0.05).

**Conclusions:**

Surgeon heart rate, salivary cortisol level, instrument usability, and Surg-TLX ratings indicate that SILC is significantly more stressful and physically demanding than the CLC. Surgeon stress and workload may impact patients’ outcomes; thus, ergonomic improvement on SILC is necessary.

Single-incision laparoscopic cholecystectomy (SILC) is a novel minimally invasive procedure to cholecystectomy and appears to have a similar safety profile as conventional laparoscopic cholecystectomy (CLC) [1–3]. Although patients prefer the cosmetic outcome of SILC over CLC [4], SILC procedure presents significant technical and workload challenges for surgeons [5]. By placing all the instruments through one incision, the single-incision procedure reduces the instruments’ range of motion, increases the collisions between the instruments, and decreases the optics and instruments’ degree of freedom [6]. These technical challenges could increase the surgeon workload related to the SILC. This high physical workload can increase the surgeons’ musculoskeletal injury risk [7–9]. Studies in a simulation setting have shown a significant decrease in task performance using SILC compared to CLC; this effect was consistent across all expertise levels [10] and with different SILC instrumentations [11]. In summary, SILC may adversely affect the surgeon’s health and performance, which may also lead to a compromise of safety for patients’ health and the health care delivery system [12, 13].

Although SILC has been compared frequently to CLC based upon patients’ primary and secondary outcomes [14], the impact of the single-port technique on surgeon workload is not yet fully understood. Limited studies have systematically measured surgeons’ operative stress and workload and compared stresses between SILC and CLC. Ergonomic studies are needed to quantify the surgeon stress and workload to identify ergonomic risk factors that may impact surgeons’ health and their career longevity [15]. The goal of this study was to compare surgeon stress and workload during a randomized controlled study for SILC and CLC in the operating room.

## Materials and methods

To evaluate differences in surgeon workload between SILC and CLC procedures, objective and subjective workload data were collected alongside a double-blind randomized controlled trial (RCT) comparing patient outcomes between SILC and CLC. All procedures were completed by one surgeon (NCT0148943).

### Randomization

Potential patients were identified from the clinical practice according to inclusion (electing cholecystectomy for symptomatic gallstone disease) and exclusion criteria for this randomized controlled trial (RCT) NCT0148943. Patients less than 18 years of age, pregnant women or prisoners/institutionalized individuals were excluded from the trial as were patients with American Society of Anesthesiology (ASA) class >3, those undergoing chronic treatment with opiates, biopsy-proven gallbladder cancer, or patients unable or unwilling to provide consent for the study. Enrolled patients were scheduled as early case of the day. Randomization occurred after anesthesia induction by computer-generated randomization stratified by age, gender, body mass index (BMI), and insulin-dependent diabetes mellitus. Patients remained blinded to the surgical procedure for 48 h postoperatively, using four identical occlusive dressings.

Surgeon workload data were collected for 48 cases. Patient factors, i.e., BMI, age, and gender, among cases were stratified and controlled as part of the RCT. Both SILC and CLC techniques were used to perform laparoscopic cholecystectomy. For SILC patients, one umbilical skin incision was used and performed manually using a TriPortTM trocar (WA58000T, Olympus, Inc.) by the surgeon. For the patients who underwent CLC procedures, three 5-mm ports and one 12-mm port (Hasson trocar) were located on the abdominal wall.

### Evaluation of surgeon workload

Surgeon stress and workload were quantified at three distinct time points during each case: pre-, intra-, and postoperatively. Preoperative time was defined as before randomization into CLC or SILC. Intraoperative time was defined as the time the cystic artery and duct were clipped. Finally, postoperative time was defined as time of skin closure.

Workload was measured using the surgery task load index (Surg-TLX) and instrument usability survey. The Surg-TLX was adapted from National Aeronautics and Space Administration’s Task Load Index (NASA-TLX) [16, 17] and was validated for distinguishing workloads in surgery [18]. In the Surg-TLX, surgeons rated six dimensions of workload, i.e., mental, physical, temporal, task complexity, situational awareness, and distractions, on visual analogue scales (VAS) where zero is “very low” and 20 is “very high.”

The instruments usability survey was adapted from Trejo et al. [19] and Beurskens et al.’s [20] work. The surgeon participant rated laparoscopic instrument usability (e.g., awkwardness and inability to perform precise motions) in three-point scale as “None,” “Slight,” and “Substantial.” Instrument usability was assessed after each procedure. For the analysis, these outcomes were categorized binomially as present (i.e., substantial and slight) or absent (i.e., none).

Surgeon heart rate was collected at the pre-, intra-, and postoperative time points. Surgeon heart rate was collected using a portable and wireless BodyGuardian Remote Monitoring System™ by Preventice^®^. Heart rate data were collected continuously throughout the procedure and sampled for 5 min (2.5 min on each side) of the three previously defined time points.

Surgeon stress hormone (i.e., salivary cortisol) levels were sampled at each time point (i.e., pre-, intra-, and postoperative) with the saliva collection aid (Salimetrics, part number 5016.02). Saliva samples were placed in dry ice immediately after sampling, and all samples were frozen (−80 °C) after the procedure. At the conclusion of the study, salivary samples from all cases were thawed, centrifuged at 3000 rpm, and the salivary cortisol batch was assayed using ELISA [21-3002].

### Data analysis

Patient characteristics, operative time, and workload were compared between SILC and CLC using the Statistical Analysis System (SAS^®^ version 9.3; SAS Institute Inc., Cary, NC), and intention-to-treat analysis was performed. Fisher’s exact test and equal variance *t* tests were used to address assumptions in variable characteristics, variance distribution, and sample size and compare differences in patients’ age, gender, and BMI. Differences in operative duration (defined as skin-to-skin time) between SILC and CLC were tested using equal variance *t* tests.

Data were categorized by time point during the surgery (i.e., pre-, intra-, and postoperatively). At the pre-, intra-, and postoperative time points, maximum heart rate (based on sample of 2.5 min around the time point) and salivary cortisol levels during SILC and CLC procedures were compared using Wilcoxon rank sum and *t* tests, as appropriate. To overcome the diurnal rhythm changes in the cortisol level, treatment-received analysis was also performed for the first cases of the day only between the SILC and CLC. In addition, differences in heart rate and cortisol levels were calculated between paired time points (e.g., pre- minus postoperative heart rate and pre- minus intraoperative heart rate) and were compared between SILC and CLC using Wilcoxon rank sum test, ANOVAs, and unequal/equal variance *t* tests as appropriate.

The impact of SILC and CLC techniques on each Surg-TLX subscale was compared using Wilcoxon rank sum tests. SILC and CLC tool usability ratings were compared using Chi-square tests.

## Results

### Patient demographics and operative time

Data on forty-eight procedures, 23 SILCs and 25 CLCs, were collected for this study. Additional ports were required for three SILC. Randomization stratified patients by age, gender, and BMI and was revealed to the surgical team after anesthesia induction for a double-blind RCT. Patient factors (age, gender, and BMI) and procedure duration (skin to skin) between the SILC and CLC groups did not differ statistically (Table 1).

**Table 1:** Mean ± standard deviation of patient factors and procedure durations for all cases (*n* = 48)

	Treatment		*p* value
	CLC (*n* = 25)	SILC (*n* = 23)	
Age	47.7 ± 18.0	47.3 ± 17.4	0.92^a^
Patient female (%)	72.0	78.3	0.74^b^
BMI	30.6 ± 6.3	30.4 ± 6.4	0.91^a^
Procedure duration (min)	73.2 ± 27.0	74.3 ± 26.2	0.89^a^

^a^Equal variance *t* test

^b^Fisher’s exact test

### Surgeon workload

#### Surg-TLX

Subjective ratings from the Surg-TLX assessment tool are summarized in Table 2. Mean workload for each Surg-TLX subscale for SILC was equal or higher than CLC. Physical demand was 89 % higher (*p* = 0.02) in SILC procedures than CLC.

**Table 2:** Medians and interquartile ranges of Surg-TLX subscales and the procedure difficulty question

Surg-TLX subscales	CLC (*n* = 25) median (IQR)	SILC (*n* = 23) median (IQR)	% Increase in SILC versus CLC	*p* value
Mental demand	28 (18, 38)	43 (28, 47)	55	0.05
Physical demand	23 (18, 28)	43 (23, 48)	89	0.02
Temporal demand	23 (18, 28)	23 (18, 33)	0	0.77
Task complexity	23 (18, 43)	38 (23, 48)	67	0.29
Situational awareness	23 (18, 43)	28 (23, 38)	22	0.35
Distractions	23 (18, 33)	28 (23, 33)	22	0.35
Surg-TLX	23 (15, 28)	35 (25, 43)	53	0.12

Minimum score = 0 (very low) and maximum score = 100 (very high)

#### Heart rate

**Fig. 1:**
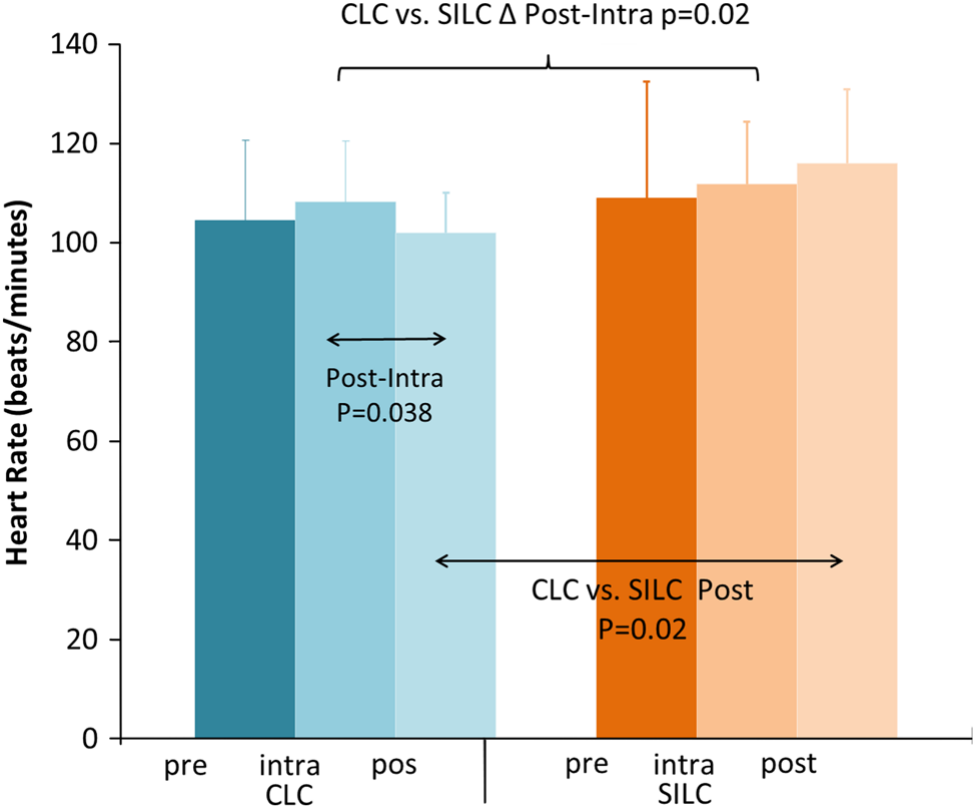
Mean and standard deviation comparisons of the maximum heart rate were within the three time points of the surgery, and between SILC and CLC. *Arrows* indicate statistical differences between SILC and CLC for specified time points, or within SILC or CLC. *Bracket* indicates significant differences between SILC and CLC for the change in the maximum heart rate

A summary of the surgeon maximum heart rate data between SILC and CLC during the three operative time points is shown in Fig. 1. Postoperative maximum heart rate was 5.74 % lower than intraoperative heart rate in the CLC procedures (*p* = 0.038). Postoperative maximum heart rate was 13.74 % higher (*p* = 0.02) in SILC than CLC. Finally, change in maximum heart rate between the postoperative and intraoperative time points was more than 100 % higher in SILC than CLC (*p* = 0.02).

#### Salivary cortisol levels

**Fig. 2:**
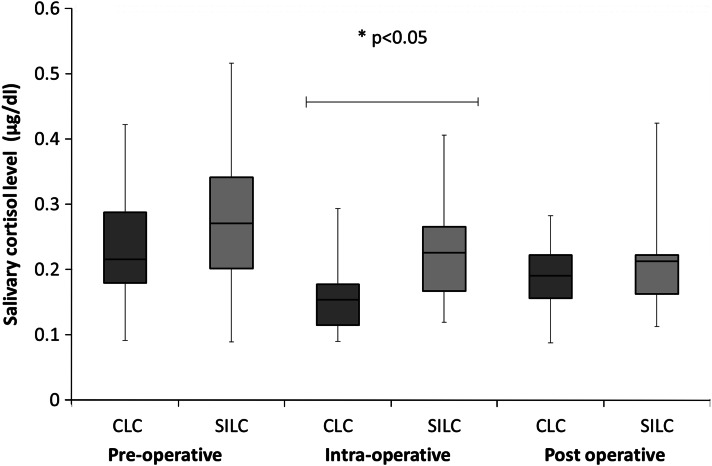
Boxplots (median, interquartile range, max, and min) of salivary cortisol levels (μg/dl) at the three time points of the surgery and between SILC and CLC. *Significant differences between SILC and CLC at specified time point

Summary of cortisol concentrations between SILC and CLC during the three operative time points is shown in Fig. 2. Intraoperative cortisol levels for the surgeon were 41.25 % higher in SILC than in CLC (*p* < 0.05).

#### Tools usability

Comparing laparoscopic instruments usability between SILC and CLC, SILC tools were more frequently reported (*p* < 0.01) to be awkward to manipulate and unable to perform precision motions (Table 3).

**Table 3:** Frequency (% of cases) with which surgeon postoperatively reported problems with laparoscopic tools usability

Usability questions	CLC	SILC	*p* value
Instruments awkward to manipulate	1 (4 %)	16 (70 %)	<0.01
Cannot perform fine/precision motions	1 (4 %)	9 (39 %)	<0.01

## Discussion

SILC improves patient satisfaction compared to CLC [21], but the impact of the SILC technique on the surgeon has not been well studied. Our results show that SILC is physically more demanding for the surgeon than CLC.

This study was conducted in parallel with a randomized controlled trial allowing us to control for patients factors and limiting surgeon bias to which patient was offered SILC. Patient’s demographics and operative time have been previously suggested to affect surgeon stress and workload; however, no significance differences between the SILC and CLC groups were observed. Previous meta-analyses found that SILC requires a significantly longer time than CLC [21, 22]. In 2014, Koca found that surgeons require longer time to complete SILC than CLC (*p* < 0.05) [23]. With our result, we believe the surgeon has overcome the learning curve of both techniques and has reached the experience level on both techniques, SILC and CLC, even before the start of this study.

SILC was associated with significantly more awkward manipulations and caused more difficulty in performing the fine and precise movements when compared to CLC. Previous studies claim that single-incision techniques are more challenging than the conventional laparoscopic technique [10, 11], because of the instruments’ collisions, the narrow external surgical space for both surgeon hands and instruments [6, 24], and the limited range of motion [25]; this study confirms these with the instruments usability survey. Although Podolsky found that TriPort (which we used in our study) had the minimal elastic recoil force when the instruments released in maximum opposition in comparison with other reduced port techniques such as single-incision laparoscopic surgery (SILS) and single-port access [26] techniques, all SILC techniques have the common constraint on degrees of freedom. In contrast, multiple-port laparoscopy involves less elastic recoil and has a greater independence of movements [27]. The physical constraints of SILC could increase the difficulty of executing fine movements during surgery [24]. Moreover, the elastic recoil associated with SILC could increase the muscular fatigue and workload [28]. Elastic recoil and one incision instrumentation make the force exerted by the instruments on the abdominal tissue of the patients in SILC greater than CLC [29, 30]; however, this was not correlated with the postoperative pain or adverse patient outcomes. The physical constraints of SILC may explain Table 3 findings that SILC tools were more frequently associated with awkward manipulations [6, 24, 31]. Awkward manipulation and lack of precise movements may have a negative impact on both surgeon and patient safety. Awkward manipulation was shown to increase surgeons’ injury risks [32], and loss of precise movements may lead to longer operative time [24].

Salivary cortisol was used as an objective physiological measure of surgeon stress during the procedures. Although variability could occur from external and internal factors that affect the salivary cortisol levels [33–35], the sources of variability were limited by including only one surgeon in this study. Considering the diurnal rhythm changes in the cortisol level, we conducted treatment-received analysis for the first cases of the day only to match the times of the samples. During the procedure, SILC resulted in significantly higher salivary cortisol levels than CLC (Fig. 2), which may indicate that SILC is more stressful than CLC. Salivary cortisol has been shown to rise with increases in mental stress [36], which could also indicate that SILC is more mentally demanding than CLC. High mental stress may decrement the surgeons’ performance [37] and decision-making ability [23, 38, 39], which in turn may increase the operative duration and surgical errors that affect patient outcomes [10].

As it is another known objective measure of stress similar to cortisol [40, 41], the maximum heart rate was recorded. The maximum heart rate was found to be significantly higher postoperatively in SILC than in CLC. In addition, the difference in the mean of the maximum heart rate and the difference between pre-incision and postoperative times, and between intraoperative and postoperative were significantly higher in SILC than in CLC (*p* = 0.01 and *p* = 0.02, respectively). For CLC, the maximum heart rate increased from the pre-assignment to the surgery to intraoperative period and then dropped significantly after the intraoperative period to the postoperative period. If we compare that to the maximum heart rate pattern in the SILC, which is increasing from preoperative point till the end of the procedure, that may indicate that CLC is less stressful than the SILC. Previous work found that stress and workload increase the sympathetic tone which increases the heart rate [42]. Our study is in line with another study using the heart rate to measure the stress in the operating room [43]. These studies corroborate our findings that CLC may be less stressful to the surgeon than SILC may be.

Surg-TLX results demonstrated that SILC is 89 % more physically demanding than CLC in a statistically significant manner. Previous studies have shown that single-incision laparoscopic surgery is more technically demanding than conventional laparoscopic surgery for the surgeon or trainees [10]. Our results were supported by previous studies in simulation settings. In 2011, Montero found that SILC has 35–53 % higher than conventional laparoscopy as demonstrated by Surg-TLX [11]. Also, Riggle et al. [44] found that SILC caused greater mental strain than conventional laparoscopy. Koca et al. [23] found that SILC had significantly higher Surg-TLX subscales than CLC (*p* ≤ 0.01) and supported his results with electromyography (EMG) data which revealed that SILC was associated with higher muscular activity for the shoulder and upper arm than CLC. Many factors could increase the perceived physical workload including the instrumentation, but the high dependency of motion of the tools and high elastic recoil internally and externally in SILC require more muscular effort from the surgeon and leads to higher required physical workload on the surgeon hand and forearm [27]. Higher physical workload with SILC may increase the surgeon’s fatigue, muscular symptoms, and injuries [7, 45]. In 2012, Morandeira-Rivas [46] found that 81 % of the survey respondents reported musculoskeletal symptoms in two or more areas during and after laparoendoscopic single-site surgery (LESS). Surgeons’ physical injuries may impact surgical productivity by increasing days of absence and decreasing years of practice for surgeons. The resultant decrease in productivity will only worsen the problem of increasing need in the surgical workforce [47, 48].

To our knowledge, this is the first study to compare surgeon stress and workload between SILC and CLC in clinical setting. Additionally, the combination of data from validated objective and subjective measures of stress and workload together in one study follows the recommendations of many reviews in the ergonomics researches in surgery [37].

One limitation of this study is the enrollment of only one surgeon. However, the single-surgeon study eliminates the interpersonal variability and allows for better workload comparison between SILC and CLC. There is bias risk in the use of the subjective questionnaires by one surgeon. This questionnaire was well validated and used in the surgical suites, and the risk of the bias was minimized by the use of the physiologic objective methods. Moreover, double blinding and randomization increase the accuracy of the preoperative heart rate and salivary cortisol measures, so we used them as baseline values. Our results apply only to the single-incision laparoscopic cholecystectomy and do not include the application of single-incision laparoscopy for other surgical specialties. Additionally, some heart rate measures are missing from this study (available heart rate data for SILC: pre-randomization = 7, intraoperative = 13, postoperative = 11; for CLC: pre-randomization = 8, intraoperative = 10, postoperative = 12). The diurnal rhythm change in the salivary cortisol is one of the limitations in any stress study. We overcome the diurnal rhythm changes by recording the time of the samples conducted a treatment-received analysis for the salivary cortisol data after we excluded non-first cases of the day (excluded cases from CLC = 9; excluded cases from SILC = 2), and the significance remained consistent.

Based on our findings, it can be concluded that workload during CLC is lower than SILC for the surgeons. The increased burden from SILC procedures on the surgeon could decrease surgical performance and/or surgeon health. Unless significant changes to the current SILC occur with further studies on the impact of these changes on surgeons and patients, alternatives to the current SILC should be considered.
